# Editorial: Viral surface spikes: host cell entry, immune responses and evasion, and implications for viral infection, inhibition and rebound

**DOI:** 10.3389/fimmu.2026.1857092

**Published:** 2026-04-30

**Authors:** Sophie M.-C. Gobeil, Maolin Lu, Amit Sharma

**Affiliations:** 1 Département de Biochimie, de Microbiologie et de Bio-informatique, Université Laval, Québec, QC, Canada; 2 Institut de Biologie Intégrative et des Systèmes, Université Laval, Québec, QC, Canada; 3 PROTEO, Le Regroupement Québécois de Recherche sur la Fonction, L’Ingénierie et les Applications des Protéines, Québec, QC, Canada; 4 Centre de Recherche en Infectiologie de l’Université Laval, Québec, QC, Canada; 5 Department of Cellular and Molecular Biology, School of Medicine, The University of Texas at Tyler Health Science Center, Tyler, TX, United States; 6 Department of Population Health and Pathobiology, College of Veterinary Medicine, North Carolina State University, Raleigh, NC, United States; 7 Comparative Medicine Institute, North Carolina State University, Raleigh, NC, United States

**Keywords:** fusion protein, immune modulation, spike, vaccine, viral entry and infection, viral evasion

Many viruses with high pandemic potential, including the severe acute respiratory syndrome coronavirus 2 (SARS-CoV-2), human immunodeficiency virus (HIV-1), and respiratory syncytial virus (RSV), rely on class I membrane fusion proteins to initiate infection (1–3). These spike proteins, often glycoproteins, facilitate viral entry by binding to host-cell receptors and mediating membrane fusion, while also triggering host immune responses such as cytokine production, B-cell activation, and antigen presentation. Their dual roles in mediating cell entry and shaping immune recognition make them prime targets for vaccines and antiviral therapies. Notable examples of such proteins include the HIV-1 envelope (Env), SARS-CoV-2 spike (S), influenza hemagglutinin (HA), and RSV fusion (F) glycoproteins. In addition to facilitating viral entry, these proteins can evade immune surveillance through mechanisms such as extensive glycan shielding (4), high mutation rates (5), antigenic drift (6), and structural variability (7, 8) – factors that complicate the development of effective vaccines and therapeutics (9). A detailed understanding of their roles in host-cell entry, immune activation, and immune evasion is essential for developing strategies to block viral infection and overcome challenges such as antibody or drug resistance and viral rebound.

This Research Topic brings together three reviews and eight original research articles that collectively examine the structural, functional, and immunological properties of viral surface spike proteins and their interplay with host cells.

The reviews provide foundational context for understanding viral entry and immune modulation. Xu et al., present an in-depth analysis of RSV molecular virology and outline the current landscape of prefusion F-targeted RSV vaccine platforms and antibody-based prophylaxis strategies. Grunst et al., compare the fusion glycoproteins of SARS-CoV-2 and HIV-1, highlighting similarities and differences in viral entry pathways and in the mechanisms by which antibody responses neutralize infection and promote clearance of infected cells. Odidika et al., examine current strategies to detect, characterize, and predict HIV-1 antibody resistance (HIVAR), with particular emphasis on broadly neutralizing antibodies (bNAbs) and the challenges HIVAR poses for their broader clinical application.

Glycan shielding is a major strategy used by viruses to evade host immune responses. Peng et al., analyze the *N*-glycosylation profiles of the recombinant spike subunit 1 (S1) protein of SARS-CoV-2 from the parental strain and several variants of concern, including Alpha, Beta, Gamma, Delta, and Lambda. Their work provides a comprehensive characterization of *N*-glycan structures and their implications for glycan shielding and immune evasion, helping to understand how glycosylation patterns influence SARS-CoV-2 infectivity and immune escape. Preclinical and clinical observations have suggested that acetylsalicylic acid (ASA) may help reduce the severity of COVID-19. Building on this premise, Perico et al., examine how ASA alters the SARS-CoV-2 S protein glycosylation and selectively reduces its binding to the ACE2 receptor. Their results demonstrate that pre-incubating the S1 subunit of the S protein with ASA diminishes binding to ACE2 on Vero cells in a dose-dependent manner.

High mutation rates represent another key strategy by which viruses evade host immune defenses, as illustrated during the COVID-19 pandemic by the emergence of numerous SARS-CoV-2 variants harboring mutations in their S protein (10). In their retrospective analysis, Peng et al., examine the evolution of the S protein between 2020 and 2024. Using the CoVFit model, they assess the virus’s adaptability and immune-escape mechanisms, offering insights to guide the development of effective public health interventions and vaccines, and to predict future viral behavior. In a similar context, VP2, the primary host-protective antigen of infectious bursal disease virus IBDV in chickens, carries the Q221K mutation among others in the newly prevalent nVarIBDV variant in China. Xiong et al., show that Q221K in VP2 constitutes part of a conserved antigenic site recognized by a new monoclonal antibody, and alterations at site 221 are associated with antigenic shifts. These studies underscore the important role of single or cumulative modifications in entry-mediating viral proteins in balancing immune evasion and viral adaptation.

Two additional SARS-CoV-2 research articles further explore molecular mechanisms of viral adaptation and potential therapeutic strategies. Cao et al., investigate the interplay between viral immune evasion and the development of therapeutic countermeasures using a computational framework. By combining molecular dynamics simulations and structural analyses, they examine the immune escape mechanisms of the SARS-CoV-2 Omicron variant and design broad-spectrum nanobodies. Using nine clinical SARS-CoV-2 isolates collected at different stages of the COVID-19 pandemic, Magnus et al., evaluate viral entry across four human cell lines with the fusion inhibitor camostat and the endocytosis inhibitor aloxistatin, correlating entry phenotypes with viral genotypes. Their findings show that SARS-CoV-2 has progressively enhanced its ability to exploit endocytic pathways while retaining TMPRSS2-dependent membrane fusion.

Immune modulation, including interferon induction, cytokine production, and B-cell activation, represents a central aspect of virus-host interaction that shapes both early viral infection and disease outcomes (11, 12). Interferon-induced transmembrane proteins (IFITMs), a class of interferon-stimulated genes, block the entry of diverse pathogenic viruses by localizing to specific cellular compartments – IFITM1 at the plasma membrane and IFITM3 in late endosomes. Prikryl et al., show that cyclosporine treatment redistributes IFITMs into intraluminal vesicles of late endosomes, relieving the viral fusion block. These findings highlight a critical role of IFITM trafficking in antiviral defense and a novel mechanism by which cyclosporine modulates cellular susceptibility to viral infection. Complementing this, Guerra-Castillo et al., examine viral coreceptor usage and immune activation in treatment-naive, late-presenting people living with HIV-1. In this cohort, most individuals harbored CCR5-tropic viruses, which were associated with higher IL-6 levels, while the less prevalent CXCR4-tropic viruses correlated with increased T-cell activation markers, and elevated IL-6 emerged as a negative predictor for CXCR4-tropic viruses. Together, these studies highlight the complex, bidirectional interplay between viral tropism, immune activation, and host antiviral defenses.

In summary, the contributions in this Research Topic offer a comprehensive view on viral entry mechanisms, immune activation and evasion, and strategies to overcome viral resistance and rebound. Advances in virology, immunology, cell biology, and structural biology are accelerating discovery in these areas. By leveraging these cutting-edge approaches, this Research Topic deepens our understanding of how viruses use surface spikes for adaptation and persistence, shedding light on virus-host co-evolution. This knowledge will help guide the development of more effective vaccines and therapeutics against globally relevant viral infections.
